# Dexmedetomidine reduces myocardial ischemia-reperfusion injury in young mice through MIF/AMPK/GLUT4 axis

**DOI:** 10.1186/s12871-022-01825-z

**Published:** 2022-09-14

**Authors:** Siyu Chen, Aimei Li, Jianjiang Wu, Yidan Huang, Tiantian Zou, Taiwangu Tailaiti, Jiang Wang

**Affiliations:** Department of Anesthesiology, The First Affiliated Hospital of Xinjiang Medical University, No.137, Liyushan Road, Xinshi District, Urumqi, Xinjiang, 830054 Uygur Autonomous Region China

**Keywords:** Dexmedetomidine (Dex), MIF/AMPK/GLUT4 axis, Heart ischemia-reperfusion injury

## Abstract

**Background:**

Reperfusion of ischemic tissue has adverse impact on the myocardium. Dexmedetomidine (Dex) is a α2-adrenergic receptor (α2-AR) agonist with sedative and analgesic effects. Macrophage migration inhibition factor (MIF) is a pressure-regulating cytokine and is responsible for inflammatory and immune diseases. This study aims to reveal the consequences of Dex on myocardial ischemia-reperfusion injury (IRI) in young mice.

**Methods:**

Fifty mice were raised and examined. At the end of the experiment, all mice were euthanized. The anterior descending department of the left coronary artery in mice was under ischemia for 60 min, then the ligation line was released and reperfused for 120 min to establish the IRI model. Mice were randomly divided into Sham, control, treatment using 4,5-dihydro-3-(4-hydroxyphenyl)-5-isoxazoleacetic acid (ISO-1), Dex treatment, and Dex combined ISO-1 treatment groups. Interleukin (IL)-6, IL-10 and tumor necrosis factor (TNF-α) were determined by enzyme-linked immunosorbent assay (ELISA). Reactive oxygen species (ROS) and ATP levels were recorded. The expressions of MIF, P-adenosine monophosphate-activated kinase α (AMPKα), glucose transporter (GLUT)4, Bax and Bcl-2 were detected by Western Blot (WB). Hematoxylin and Eosin (H&E) staining was used to study cell morphology. Apoptosis was detected by terminal deoxynucleotidyl transferase dUTP nick end labelling (TUNEL) assay. Echocardiography was carried out at the end of reperfusion, and the infarct size was calculated by Electron microscopy.

**Results:**

I/R + Dex group showed significantly increased IL-6 and TNF-α levels and reduced myocardial cell necrosis and apoptosis. H&E staining showed alleviated myocardial disorder, myocardial cell swelling, myocardial fiber fracture, and inflammatory cell infiltration in I/R + Dex group. Myocardial cell necrosis and apoptosis were significantly reduced in I/R + Dex group. ATP level in myocardial tissue of mice in I/R group was substantially decreased, while that in Dex group was increased. WB results showed that MIF, P-AMPK α, GLUT4 and Bcl-2 levels were increased and Bax levels were decreased in I/R + Dex group.

**Conclusion:**

Dex may exert myocardial protection in young mice through MIF/AMPK/GLUT4 axis.

**Supplementary Information:**

The online version contains supplementary material available at 10.1186/s12871-022-01825-z.

## Introduction

The main clinical manifestation of ischemic heart disorder (IHD) is acute myocardial infarction (AMI) [1], whose prognosis improves with the timing of revascularization. Paradoxically, myocardial reperfusion may be harmful because ischemia-reperfusion injury (IRI) is an oxidation-driven process that can damage other organs [2, 3]. At the same time, reperfusion of ischemic tissue can also cause irreversible harm to the myocardium, which is called cardiac ischemia reperfusion injury (IRI) [4]. This can lead to morbidity and mortality from cardiac intervention after a heart assault or stroke [5, 6], as well as different pathological conditions such as acute kidney injury, muscle injury, organ transplantation, hypovolemic shock and optionally available surgery [5, 7].

Dexmedetomidine (Dex) is a α2-adrenergic receptor (α2-AR) agonist with sedative and analgesic effects [8, 9]. At the same time, it also has cardioprotective effect and can reduce cardiac iron death and septic heart injury caused by sepsis. Macrophage migration inhibition factor (MIF) is a pressure-regulating cytokine acting as multiple roles in many inflammatory and immune diseases [10–12]. MIF regulates the activation of inflammatory cells and the launch of different pro-inflammatory cytokines [13], and its pathological effects have been stated in many inflammatory diseases such as rheumatoid arthritis, atherosclerosis, sepsis and cardiovascular disease [14, 15]. It has also been proven to prevent the heart from undergoing myocardial (IRI). However, the correlation between Dex and MIF and the mechanism of action remain unclear.

Glucose metabolism depends on glucose uptake by cells. However, glucose cannot enter cells freely via the lipid bilayer of cell membrane, and glucose uptake can only be carried out by means of glucosetransporters (GLUT) on cell membrane. One such protein, glucose transporter4 (GLUT4), is found in fat and muscle tissue. Study has shown that the regulation of GLUT4 in muscle requires the participation of adenosine monpophosphate-activated kinase (AMPK) [16] but its exact mechanism is unclear.

In this study, we installed the cardiac IR model of male C57BL/6 mice by ligation of the left coronary artery. The objective is to find out about the impact of Dex on cardiac IR and its molecular mechanism.

## Materials and methods

### Mice

This study was approved by the Animal Experimental Medical Ethics Committee of our hospital (IACUC-20170214025) and carried out in accordance with ARRIVE guidelines. All laboratory procedures were in accordance with the *Guidelines for the Care and Use of Laboratory Animals*. Non-pathogenic C57BL/6 mice (male; 8-week-old; 18-20 g) were purchased from Beijing Weitonglihua Experimental Animal Technology Co., LTD. (Production license No. SCXK (Beijing) 2016–0010) and raised in SPF Laboratory our university. At the end of the experience, all mice were euthanized.

### Myocardial ischemia/reperfusion (I/R)

The mice were fed for 1 week, and the follow-up operations were carried out after adaptation to the environment. Briefly, mice were anesthetized with the aid of intraperitoneal injection of 1% pentobarbital sodium (60 mg/kg), intubated by tracheal intubation, and ventilated by small animal ventilator. Microscopically, the anterior descending department of the left coronary artery was seen. A 6–0 silk suture was inserted 1-2 m below the root of the left atrial appendage, and the left margin of the pulmonary conus was removed. The stitching direction was parallel to the lower edge of the left atrial appendage. The mark of successful ligation was weakened movement of the myocardium tissue around that anterior wall of left ventricle and epex. In mice, the left anterior descending department was under ischemia for 60 min and reperfused for 120 min. Mice were randomized into Shan, control, treatment using treatment using 4,5-dihydro-3-(4-hydroxyphenyl)-5-isoxazoleacetic acid (ISO-1, 35 mg/kg), Dex (20 μg/kg) treatment and Dex (20 μg/kg) combined ISO-1 (35 mg/kg) treatmemt groups.

### Enzyme-linked immunosorbent assay (ELISA)

The blood samples of each group were accumulated and centrifuged (5000 g, 10 min, 4 °C) to separate the serum for Interleukin (IL)-6(cat. no. 70-EK206/3–96; MultiSciences), IL-10 (cat. no. 70-EK210/4–96; MultiSciences) and tumor necrosis factor (TNF)-α (cat. no. 70-EK282/3–96; MultiSciences) testing. These kits were used according to the operation manual.

### Cell reactive oxygen species (ROS) detection

Fresh isolated left ventricular myocardial tissue was ground into single cell suspension, 2 ml phosphate-buffered saline (PBS) and 2ul 2′-7’dichlorofluorescin diacetate (DCFH-DA) were added. The cells were incubated at 37 °C for 30 min, centrifuged at 1000 rpm for 5 min, then the cells were accumulated and washed twice with PBS. After centrifugation, the cells were collected and precipitated for fluorescence detection. The wavelength was 525 nm.

### Adenosine triphoshpate (ATP) content analysis

As previously described [17], ATP content was quantified by measuring the luminescence produced by the ATP-dependent luciferase bioluminescence assay. Left ventricular myocardial tissue was cryopreserved at − 80 °C and thawed immediately prior to automatic addition of the luciferase mixture. The protein was quantified in accordance with the directions of the ATP detection kit (cat. no. A095–1; Nanjing Jiancheng Technology Co., LTD), and detected at 636 nm by spectrophotometer. In order to reduce potential differences between samples, three replicates and analyses were performed.

### Western blotting (WB)

Mouse left ventricular myocardial tissue samples were milled in RIPA buffer and centrifuged at 12000 rpm at 4 °C for 15 min. The supernatant was accumulated for total protein analysis, and the protein concentration was decided by bicinchoninic acid assay (BCA) method. An equivalent amount of protein (30 μg) was extracted from mouse heart homogenate, separated by 10% sodium dodecyl sulfate-polyacrylamide gel electrophoresis (SDS-PAGE), and transferred to PVDF membrane. It was then sealed with 5% (W/V) skim milk at room temperature (RT) for 1 h, and incubated overnight with primary antibody at 4 °C. Primary antibodies are as follows: against Bax (cat. no. ab32503; Abcam), Bcl-2 (cat. no. ab59348; Abcam), Phospho-AMPKα (cat. no. 2535S; Cell Signaling Technology, Inc.), GLUT4(cat. no. PA5–23052; Thermo Fisher Scientific), MIF (cat. no. ab65869; Abcam) and beta-Actin (cat. no. 100166-MM10; Sino Biological) were used at the dilution 1:1000. After washing with Tris-buffered saline with Tween detergent (TBST) three times for 10 min each, the membrane strips were incubated at RT with a 1:5000 dilution of an anti-rabbit IgG (cat. no. ab205718; Abcam) or anti-mouse IgG (cat. no. ab205719; Abcam) secondary antibody conjugated to horseradish peroxidase for 1 h. Protein bands were detected by Chemiscope 3000 (Shanghai, China) and the images were quantified with the use of ImageJ version 1.51 software (National Institutes of Health).

### Hematoxylin and eosin (H&E) staining

Mouse hearts were fixed with 4% paraformaldehyde at RT overnight, embedded in paraffin, and sliced into 4 μm thick sections. At RT, the tissues were stained via hematoxylin (cat. no. CTS-1097; Fuzhou Maixin Biotechnology Development Co. LTD) for 5 min and eosin (cat. no. ZLI-9613; Beijing Zhongshan Jinqiao Biotechnology Co., LTD) for 1 min. The slides were visually examined under a microscope (cat. no. E200; Nikon; Japan).

### Apoptosis

Terminal deoxynucleotidyl transferase dUTP nick end labelling (TUNEL) staining was used to assess apoptosis. The cells were immobilized in 4% paraformaldehyde at RT for 1 h and then treated with 0.5% TritonX-100 for 10 min. After washing with PBS, cells were incubated with cell death detection kits (cat. no. MK1025; BOSTER) in accordance with manufacturer’s instructions. The nuclei were stained with 0.1 g/mL DAPI (cat. no. D1306; Invitrogen) for 5 min.

### Echocardiography

All mice were anesthetized with isoflurane (American veterinary anesthesia system) and detected through ultrasonic electrocardiogram (visualsonics VEVO 770 system, Canada) 40 MHz frequency scraper before death. The parameters of heart rate (HR), left ventricular end diastolic diameter and left ventricular end systolic diameter were collected. Then, the heart rate (HR), stroke volume (SV), ejection fraction (EF), fractional shortening (FS), cardiac output (CO), LVPWs (Left ventricle posterior wall thickness in systole), LVPWd (Left ventricle posterior wall thickness in diastole) and A (Aorta) were calculated as previously described to evaluate cardiac function [18].

### Electron microscopy and ultrastructural study

Histological and ultrastructural research were carried out using the methods previously described by Loh et al [19]. Morphological changes were observed, and images were taken using an electron microscope (Leica, Germany).

### Statistics

All statistical analyses of the data were processed in blind using Prism 8.0 software (GraphPad, San Diego, CA, USA). Data from each group were expressed as mean ± standard deviation (SD) and characterized by one-way ANOVA or Student’s t-test (and nonparametric test) for multiple comparisons. The behavioral records were statistically analyzed by two-way variance evaluation of double comparison. *P* < 0.05 was regarded statistical significant.

## Results

### Effects of Dex on serum inflammatory factor levels

**Fig. 1:**
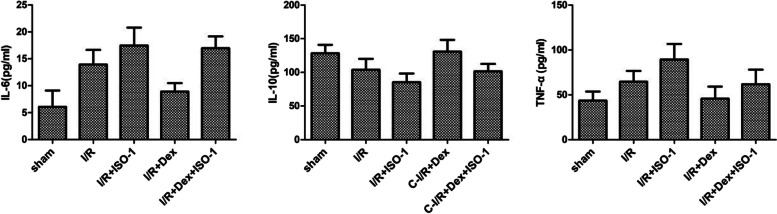
Serum inflammatory factors were detected by ELISA

The effect of Dex on serum inflammatory cytokines was measured by ELISA. The outcomes confirmed that the production of IL-6 and TNF-α in I/R + Dex group was substantially decrease than that in Sham group (*P* < 0.05; Fig. 1 and Table 1), IL-10 production was considerably elevated (*P* < 0.05; Fig. 1 and Table 1). Further addition of an MIF inhibitor (ISO-1) substantially counteracted the effect of Dex on inflammatory levels.

**Table 1:** Analysis of serum IL-6, IL-10 and TNF-α levels in each group ($\overline{x}\pm s$)

Group	IL-6(pg/ml)	IL-10(pg/ml)	TNF-α(pg/ml)
**Sham**	6.101 ± 3.020	128.350 ± 12.493	43.596 ± 10.053
**I/R**	13.930 ± 2.722^△^	103.836 ± 16.310^△^	64.519 ± 12.036^△^
**I/R + ISO-1**	17.465 ± 3.330^△▲^	85.482 ± 12.824^△▲^	89.267 ± 17.460^△▲^
**I/R + Dex**	8.932 ± 1.558^△▲▽^	130.984 ± 17.319^▲▽^	45.749 ± 13.334^▲▽^
**I/R + Dex + ISO-1**	16.960 ± 2.195^△▲▼^	101.534 ± 11.013^△▽▼^	61.667 ± 16.406^△▽▼^

^△^*P* < 0.05 vs. Sham group; ^▲^*P* < 0.05 vs. I/R group; ^▽^*P* < 0.05 vs. I/R + ISO-1 group; ^▼^*P* < 0.05 vs. I/R + Dex group

### Effect of Dex on myocardial histopathology in mice

**Fig. 2:**
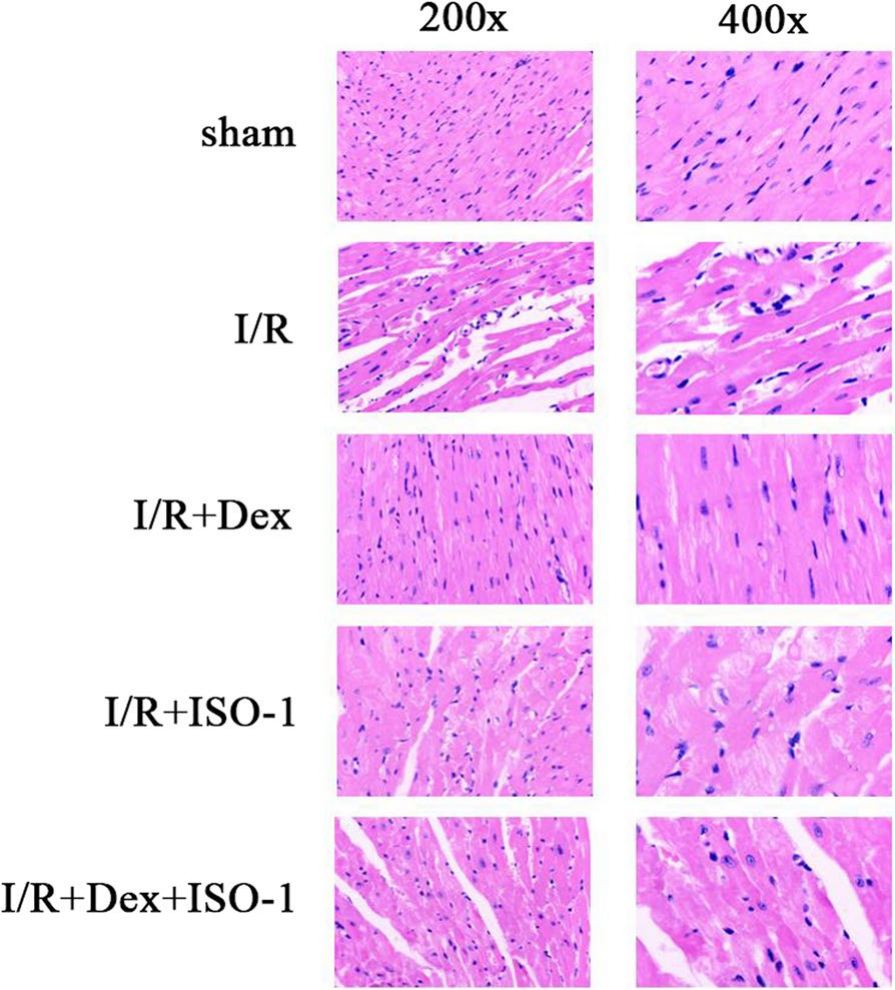
H&E staining results of mouse myocardium

H&E staining was used to assess histopathology based on morphology. In the I/R group, myocardial tissue was disordered, myocardial cells were swollen and vacuolated, some myocardial fibers were broken, and a few chronic inflammatory cells were infiltrated. Compared with I/R group, myocardial disorder, myocardial cell swelling, myocardial fiber fracture and inflammatory cell infiltration were reduced in I/R + Dex + ISO-1 and I/R + Dex groups. The lesion of I/R + Dex group was slightly milder than that of I/R + Dex + ISO-1 group (Fig. 2).

### Effect of Dex on myocardial apoptosis

**Fig. 3:**
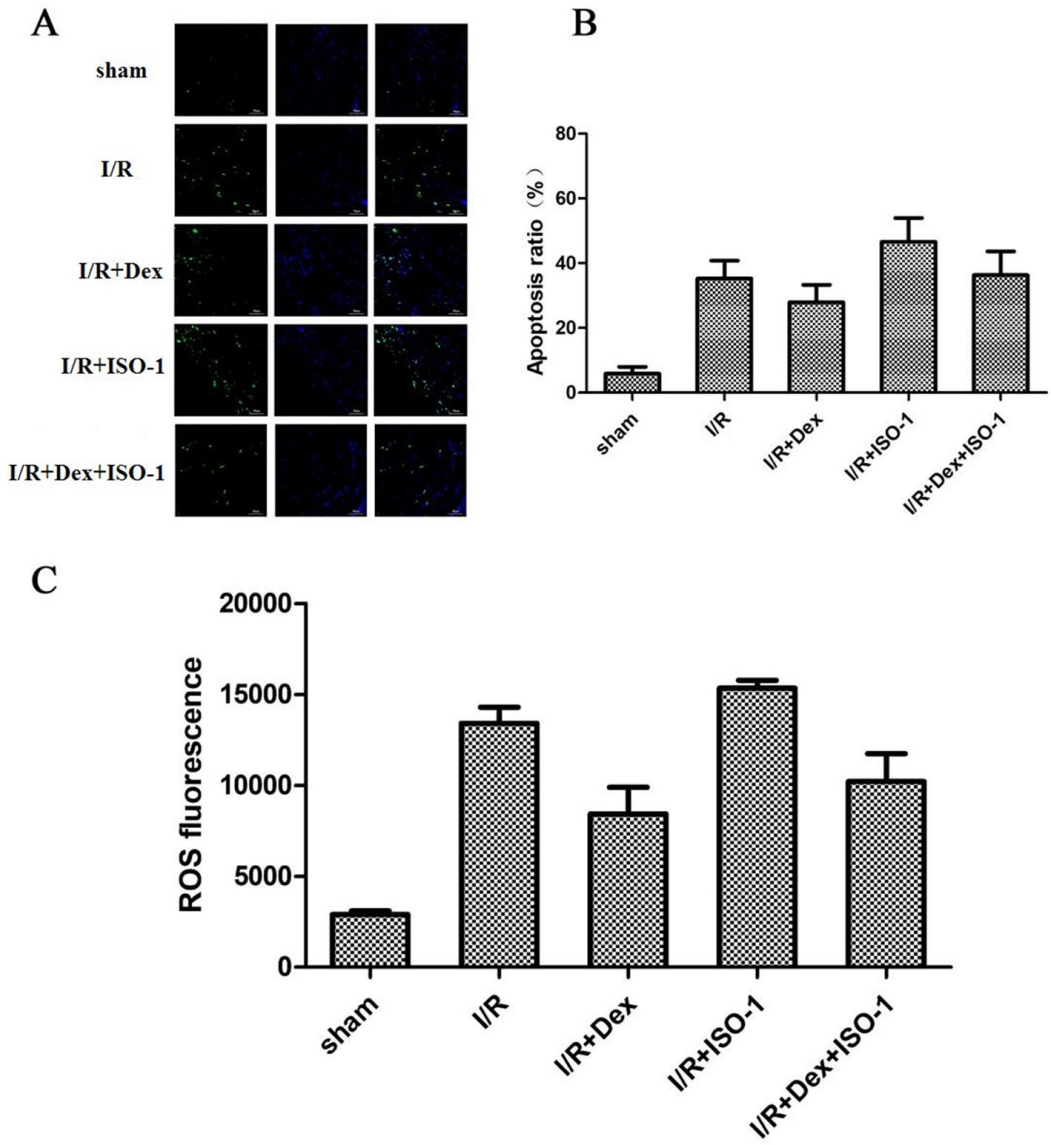
Apoptosis was detected by TUNEL and ROS. **A**, **B** Representative images of TUNEL staining showing DEX inhibits apoptosis of cardiomyocytes. **C** Representative ROS images also showed that DEX inhibited myocardial cell apoptosis

Immunofluorescence assay confirmed that in contrast with Sham group, myocardial cell necrosis and apoptosis were extensively enhanced in I/R group (*P* < 0.05; Fig. 3A, B). However, in contrast with the I/R group, necrosis and apoptosis in mice treated with Dex significantly decreased (*P* < 0.05). In addition, in comparison to the I/R + Dex group, cell death was significantly increased in the I/R + ISO-1 and I/R + Dex + ISO-1 groups, respectively (all *P* < 0.05; Fig. 3A, B). ROS detection also displayed similar trend and confirmed the above results (Fig. 3C).

### Effect of Dex on ATP content in the heart

**Fig. 4:**
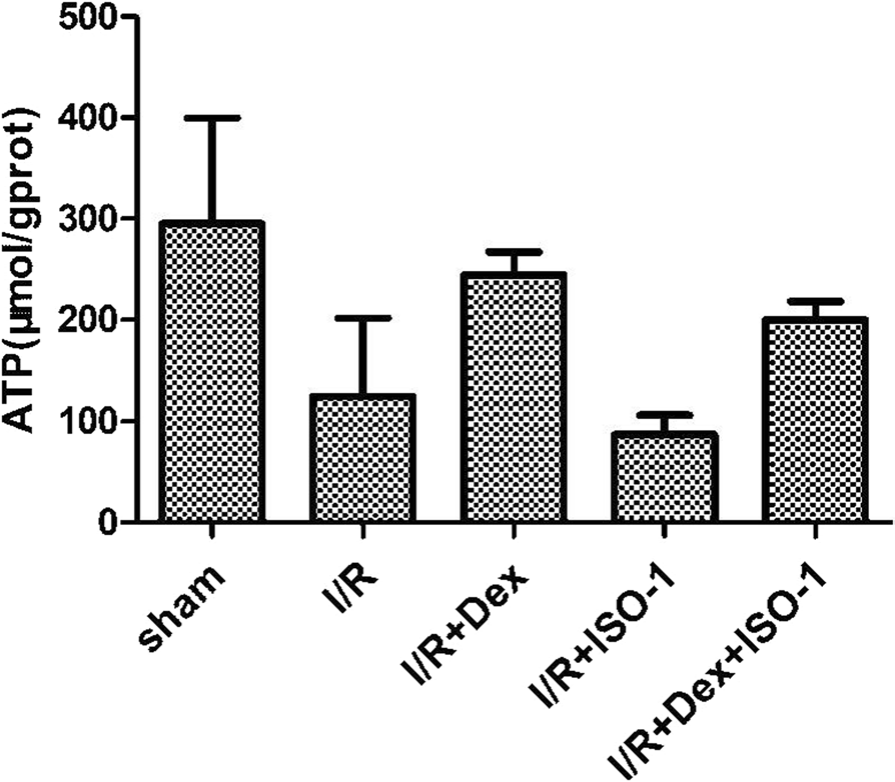
ATP levels in each group

Compared with the Sham group, ATP levels in the myocardial tissue of mice in the I/R group were significantly decreased, but significantly increased after Dex treatment. ATP content in the I/R + ISO-1 group was lower than that in the I/R + ISO-1 group, and the situation improved after Dex combined with ISO-1, but ATP level was lower than that in the I/R + Dex group (Fig. 4).

### Effects of Dex on ultrasound and evaluation of ultrastructure in mice

**Fig. 5:**
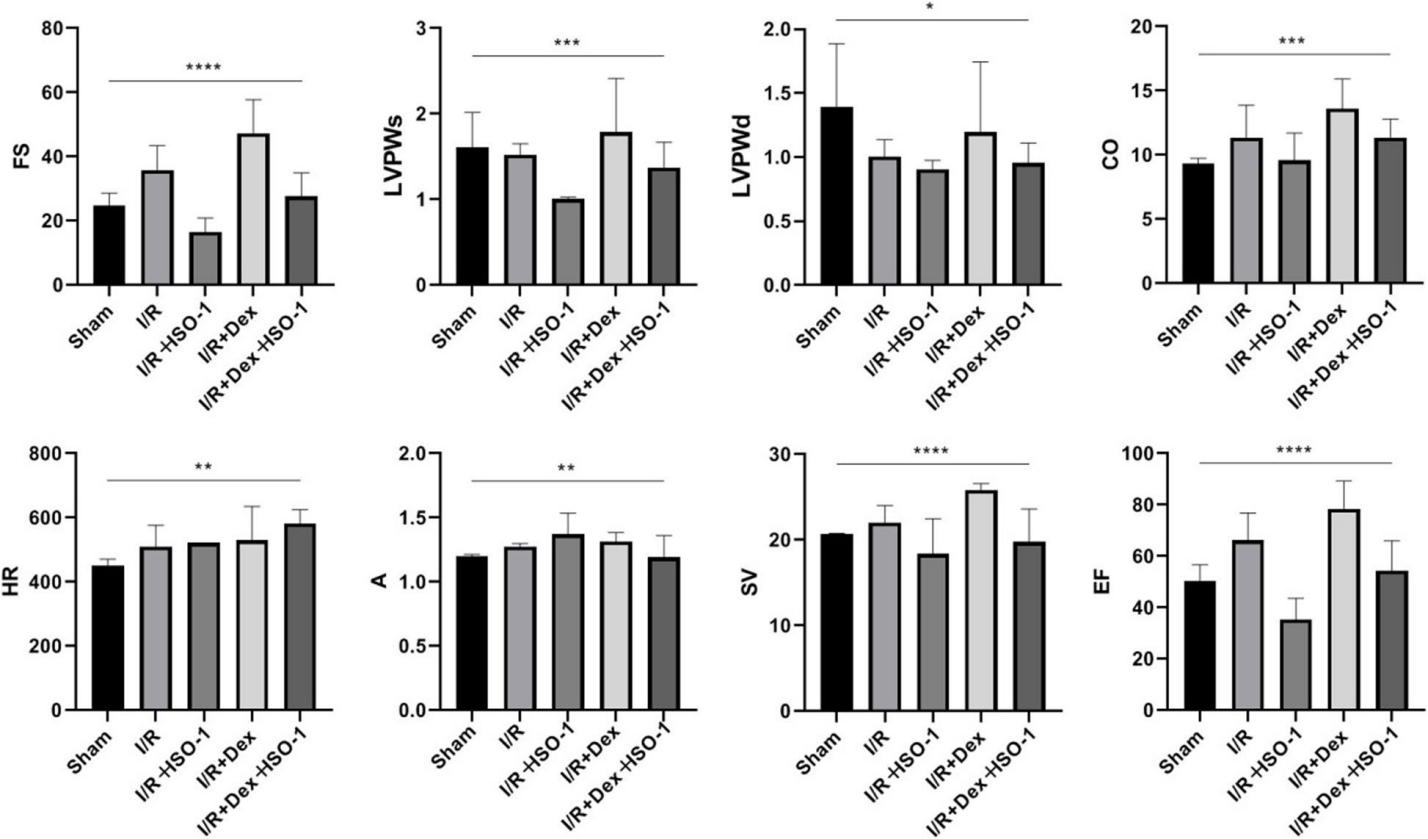
Representative image of mouse cardiac ultrasound

**Fig. 6:**
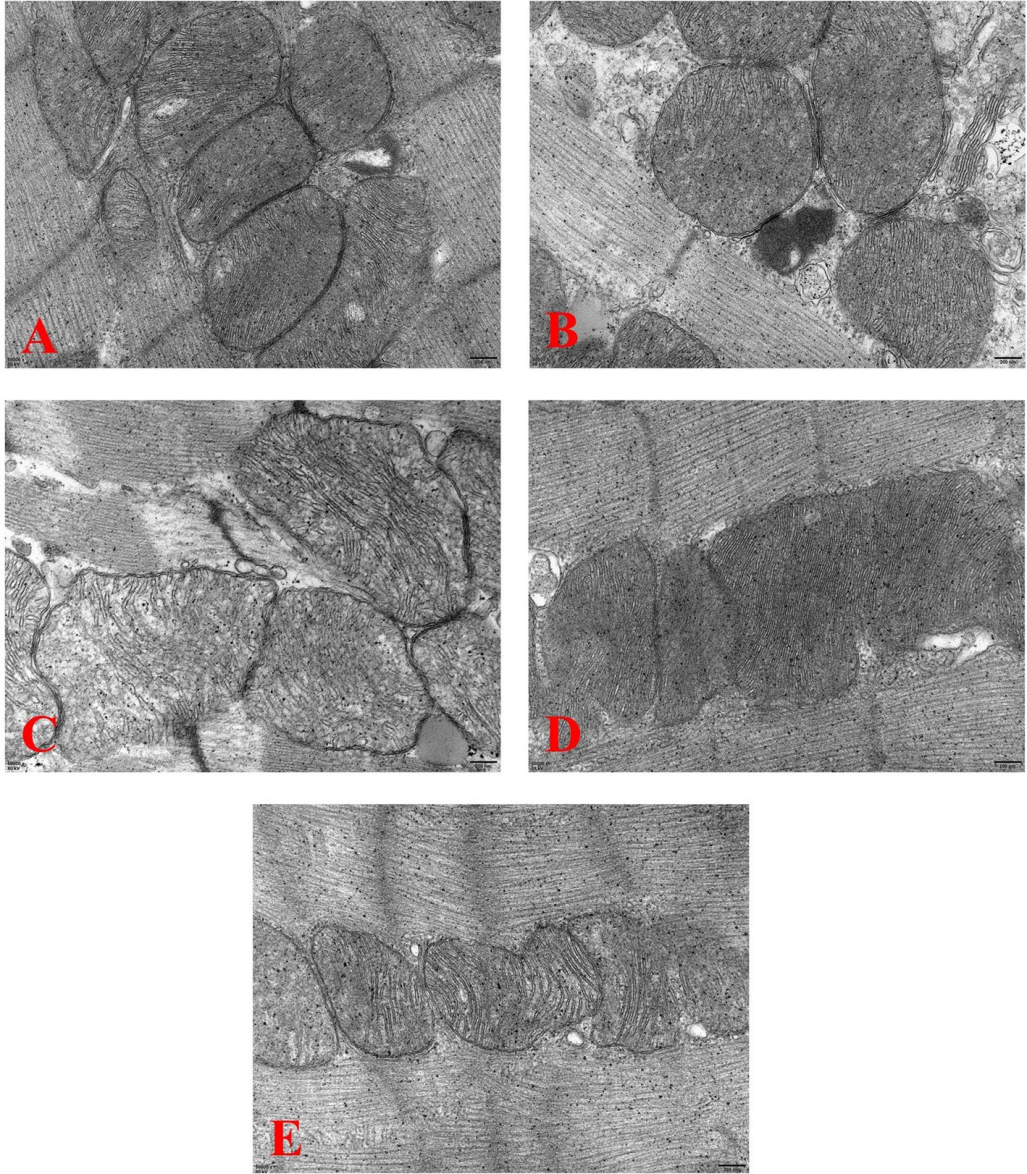
Electron microscopy of representative mouse myocardial sections in different experimental groups (200 nm). **A**: sham group; (**B**): the I/R group; (**C**): the I/R + ISO-1 group; (**D**): the I/R + Dex group; (**E**): the I/R + Dex + ISO-1 group

SV, EF, FS, CO and LVPWs were drastically expanded in the I/R + DEX group by cardiac ultrasound, but decreased in combination with ISO-1 (Table 2 and Fig. 5). Figure 6 shows myocardial electron microscopy images of each group. Electron micrographs of mice in the sham group showed normal cardiac structures (Fig. 6A). The I/R group showed extensive muscle necrosis, loss of lipid droplets, and giant destruction of myofilaments and Z-band structures (Fig. 6B). Similar adjustments were discovered in the ISO-1 group alone (Fig. 6C). However, the ultrastructure of I/R + Dex group and I/R + Dex + ISO-1 group were normal without swelling and vacuole formation (Fig. 6D, E).

**Table 2:** Effects of different intervention groups on the results of echocardiography in young mice ($\overline{x}\pm s$, *n* = 9)

Group	HR	SV	EF	FS	CO	LVPWs	LVPWd	A
**Sham**	451.34 ± 19.67	20.71 ± 4.21	50.33 ± 6.41	24.85 ± 3.77	9.35 ± 0.39	1.61 ± 0.41	1.39 ± 0.49	1.19 ± 0.15
**I/R**	509.85 ± 66.81	22.02 ± 2.01	66.32 ± 10.58	35.84 ± 7.62	11.35 ± 2.52	1.52 ± 0.13	1.01 ± 0.13	1.28 ± 0.22
**I/R + ISO-1**	521.41 ± 36.62	18.35 ± 4.13	35.42 ± 8.20	16.61 ± 4.31	9.57 ± 2.15	1.01 ± 0.05	0.91 ± 0.07	1.37 ± 0.16
**I/R + Dex**	529.94 ± 75.85	25.82 ± 2.77	78.46 ± 10.86	47.28 ± 6.53	13.62 ± 2.32	1.79 ± 0.62	1.19 ± 0.15	1.32 ± 0.21
**I/R + Dex + ISO-1**	582.19 ± 42.68	19.75 ± 3.86	54.46 ± 11.63	27.64 ± 7.31	11.35 ± 1.44	1.37 ± 0.29	0.95 ± 0.16	1.19 ± 0.17

Abbreviations: *HR* heart rate, *SV* stroke volume, *EF* ejection fraction, *FS* fractional shortening, *CO* cardiac output, *LVPWs* Left ventricle posterior wall thickness in systole, *LVPWd* Left ventricle posterior wall thickness in systole, *A* Aorta

### Dex protects myocardial IRI through MIF/AMPK/GLUT4 NF-κB axis

**Fig. 7:**
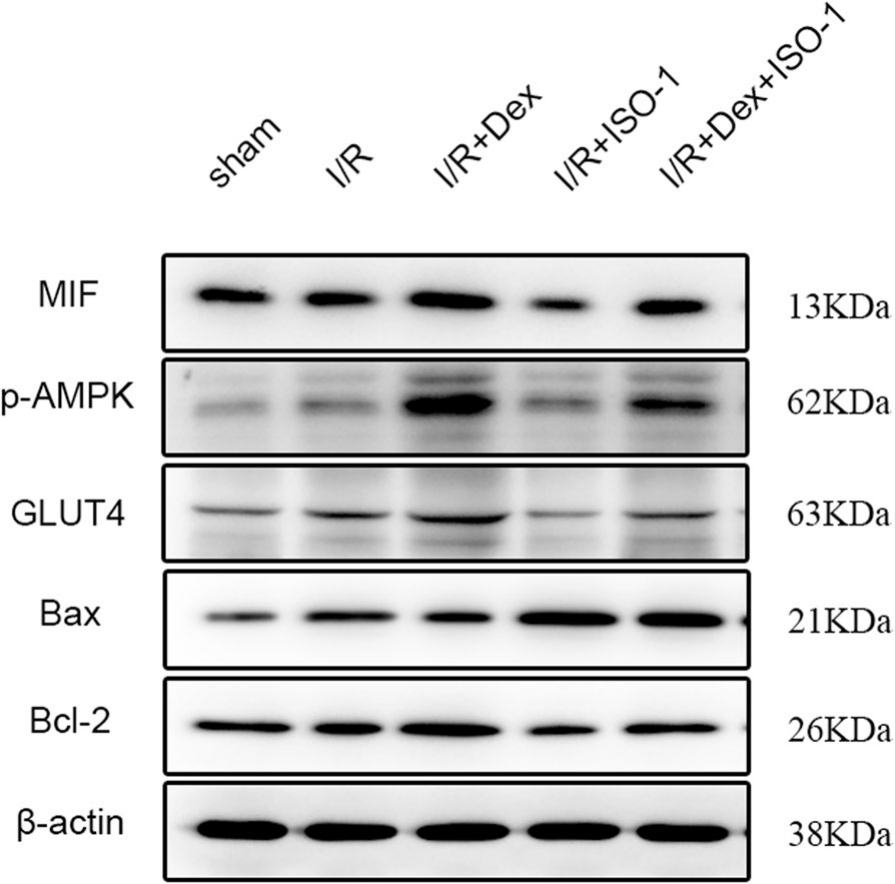
Dex protects myocardial IRI through MIF/AMPK/GLUT4 NF-κB axis

Western blotting confirmed that in contrast with the I/R group, p-AMPKα, MIF, GLUT4 and Bcl-2 protein expressions were extensively elevated in the I/R + Dex group (*P* < 0.05), Bax protein was drastically diminished (*P* < 0.05; Fig. 7).

## Discussion

IR damage causes distal organ damage, which effects in the release of reactive oxygen species (ROS) and inflammation-related molecules from ischemic tissues [20–22]. Tissue damage caused by ischemia is a major cause of fatal diseases such as heart attack and stroke [23]. Myocardial ischemia (MI) is reported to be one of the most vital danger elements for adverse cardiac outcomes in surgical patients with cardiovascular disease [24]. Reperfusion therapy is considered as one of the most effective method to preserve ischemic myocardium after AMI. However, restoring blood flow may lead to myocardial ischemia-reperfusion injury (MIRI) [25]. MIRI is an important issue affecting the prognosis of patients with myocardial infarction. The pathogenesis of MIRI often includes inflammatory response, apoptosis, calcium overload and the production of oxygen free radicals [26]. It can induce myocardial coma, no regurgitation, reperfusion arrhythmia, and even irreversible myocardial cell death [27]. Importantly, severe IRI to the heart can lead to everlasting incapacity or death [28]. Reperfusion injury is inevitable due to production of ROS and apoptosis of cardiomyocytes [29]. However, our study showed that Dex inhibited ROS production and myocardial cell apoptosis, which could prevent reperfusion injury to a certain extent. It has been reported that neutrophils and macrophages release ROS due to IR injury. These free radicals may lead to lipid peroxidation of cell membranes, increase in excitatory amino acids, and destruction of nucleic acids and enzymes [30].

Dex is a potent α2-AR agonist that is clinically appropriate for sedation in patients undergoing intubation and ventilator use during intensive care treatment. Currently, Dex is increasingly being introduced into the perioperative care of patients undergoing surgery [31]. In animal studies, Dex reduces organ damage. In clinical studies, Dex has been associated with improved heart surgery and transplant outcomes [31]. It has also been suggested that post-treatment of Dex leads to a significant reduction in concentration-dependent infarct size [32]. Kip G et al. [33] found that Dex could protect lung damage after myocardial ischemia-reperfusion in diabetic rats. Meanwhile, Dex has been found to have myocardial protection against hypertensive hypertrophic myocardial IRI [34]. Zhao et al. [35] found that Dex alleviated cerebral IRI in rats by inhibiting c-un N-terminal Kinase (JNK) pathway. Moreover, Dex reduces Nod-like receptor family pyrin domain containing 3 (NLRP3) through adenosine monophosphate-activated kinase (AMPK), thereby improving NLRP3 inflammasome activation in alveolar macrophages mediated by renal ischemia-reperfusion [36]. Dex alleviates endotoxin-induced acute kidney damage via enhancing autophagy and inhibiting NLRP3 inflammatory body activation through the 2-AR/AMPK/mammalian target of rapamycin (mTOR) pathway [37]. Similar to these studies, our study found that Dex protected myocardial cells from IRI through the MIF/AMPK/GLUT4 axis.

Cardiac IR triggers a massive inflammatory response, exacerbating cardiac damage and dysfunction. It has been reported that MIF expression is enhanced after ischemic heart injury in animal models [38]. A clinical research performed by Yu et al. also confirmed that plasma MIF level was intently associated with the severity of myocardial injury [39]. Activation of the MIF-AMPK pathway has been found to increase glucose transporters and glucose metabolism, reducing IRI [40]. In addition, the shielding role of MIF in acute kidney injury after cardiac surgery has been investigated [41]. Several research have reported the protective function of MIF in cardiac IR injury, including promoting glucose uptake through AMPK activation [42], inhibiting oxidative stress [43] or inhibiting JNK-mediated apoptosis [44]. Our study also found that ROS and myocardial cell apoptosis increased after inhibition of MIF, indicating that MIF could protect myocardium from reperfusion injury.

There are some limitations to this study. Although Dex has been shown in vivo to be succesful of lowering myocardial ischemia-reperfusion damage in young mice through MIF/AMPK/GLUT4 axis, it has not been tested in vitro and further studies are needed.

## Conclusion

Dex reduces myocardial IRI in young mice by MIF/AMPK/GLUT4 axis. Future research evaluating the cardioprotective role of Dex in the scientific putting of MIRI is necessary.

## Supplementary Information

**Additional file 1.**

## Data Availability

The data used and analyzed during the current study are available from the corresponding author on reasonable request.

## Abbreviations

Dex: Dexmedetomidine
α2-AR: α2-adrenergic receptor
MIF: Macrophage migration inhibition factor
IRI: Ischemia reperfusion injury
WB: Western Blot
IHD: Ischemic heart disorder
AMI: Acute myocardial infarction
IR: Ischemia reperfusion
GLUT: Glucosetransporters
GLUT4: Glucosetransporter 4
ELISA: Enzyme-linked immunosorbent assay
ROS: Reactive Oxygen Species
RT: Room temperature
H&E: Hematoxylin and eosin
HR: Heart rate
SV: Stroke volume
EF: Ejection fraction
FS: Fractional shortening
CO: Cardiac output
LVPWs: Left ventricle posterior wall thickness in systole
LVPWd: Left ventricle posterior wall thickness in diastole
A: Aorta
MI: Myocardial ischemia
MIRI: Myocardial ischemia-reperfusion injury
