# Supplementary figures and images for: Dexmedetomidine reduces myocardial ischemia-reperfusion injury in young mice through MIF/AMPK/GLUT4 axis
Source: BMC Anesthesiol. 2022 Sep 14;22:289. doi: 10.1186/s12871-022-01825-z (PMC9472426; doi:10.1186/s12871-022-01825-z)

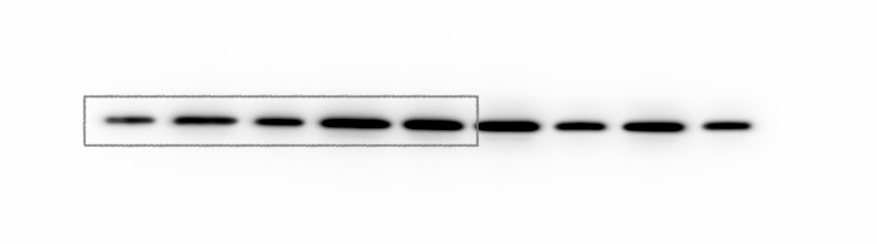

Supplement: Supplementary file 1 — Additional file 1. [file 12871_2022_1825_MOESM1_ESM.zip › Supplementary/1-Bax- original (mice).tif]

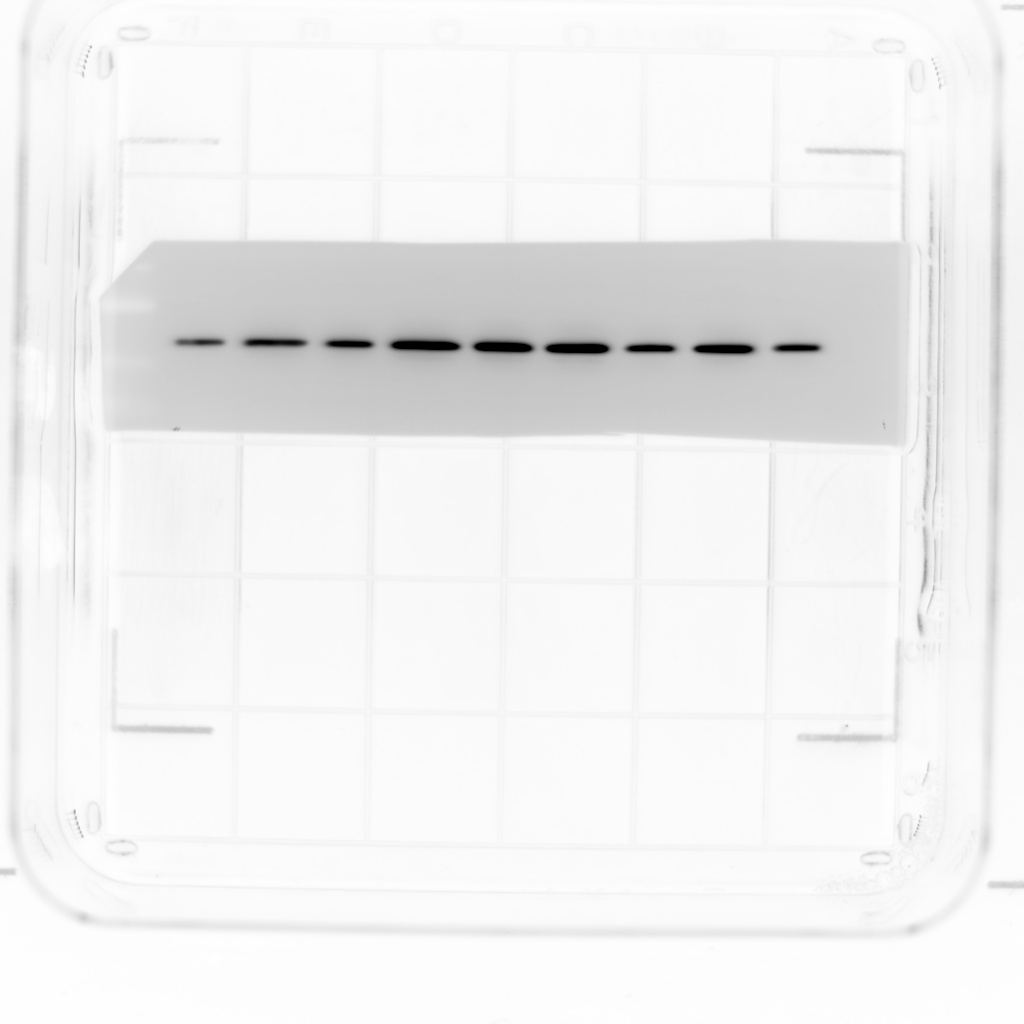

Supplement: Supplementary file 1 — Additional file 1. [file 12871_2022_1825_MOESM1_ESM.zip › Supplementary/1-BAX.jpg]

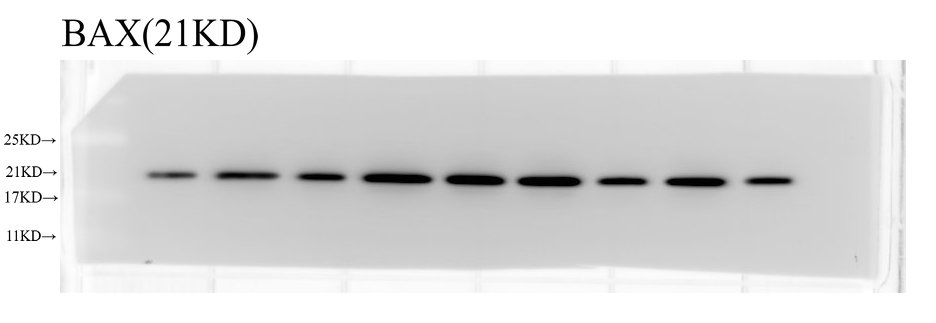

Supplement: Supplementary file 1 — Additional file 1. [file 12871_2022_1825_MOESM1_ESM.zip › Supplementary/BAX-marking.jpg]

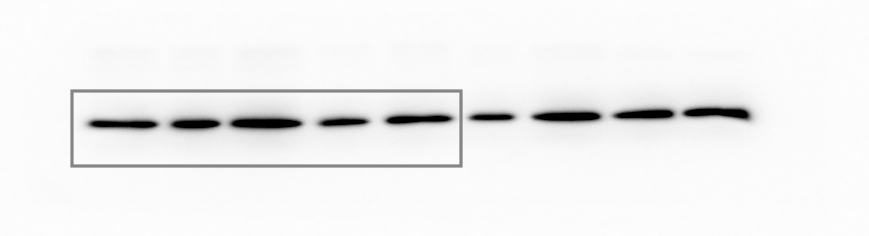

Supplement: Supplementary file 1 — Additional file 1. [file 12871_2022_1825_MOESM1_ESM.zip › Supplementary/Bcl-2 -original (mice).tif]

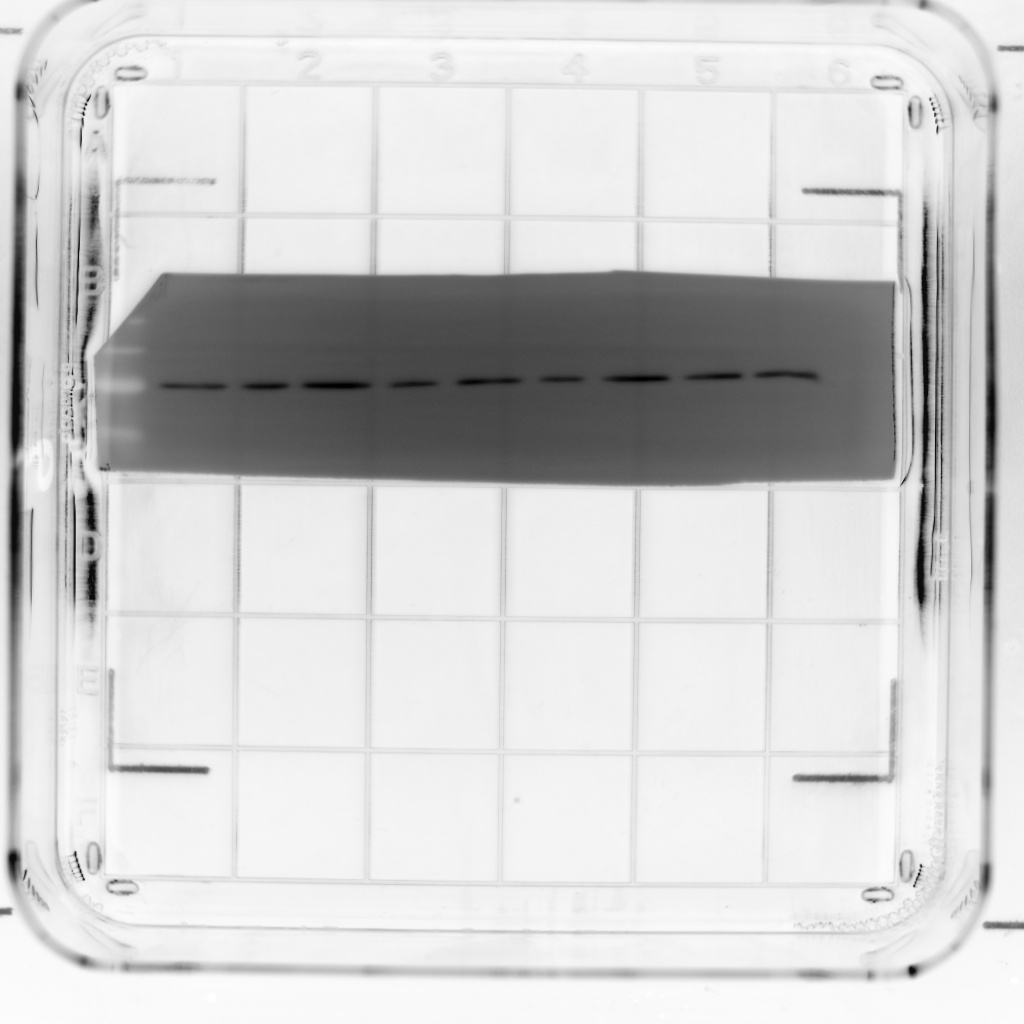

Supplement: Supplementary file 1 — Additional file 1. [file 12871_2022_1825_MOESM1_ESM.zip › Supplementary/BCL-2.jpg]

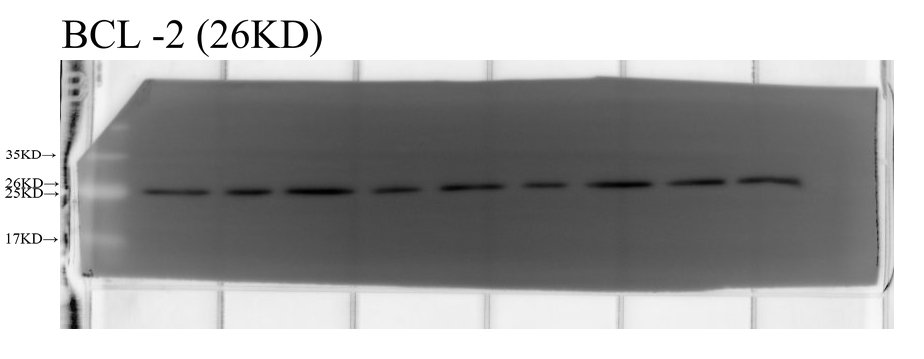

Supplement: Supplementary file 1 — Additional file 1. [file 12871_2022_1825_MOESM1_ESM.zip › Supplementary/BCL-2-marking.jpg]

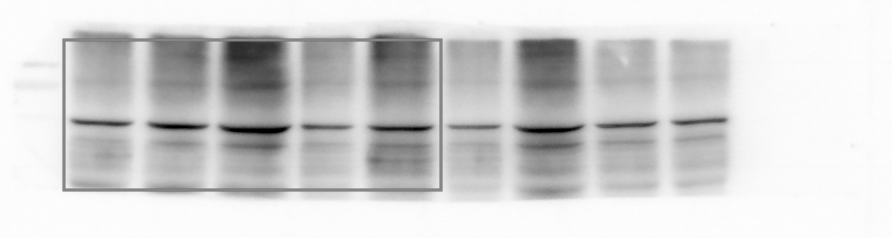

Supplement: Supplementary file 1 — Additional file 1. [file 12871_2022_1825_MOESM1_ESM.zip › Supplementary/GLUT4 -original (mice).tif]

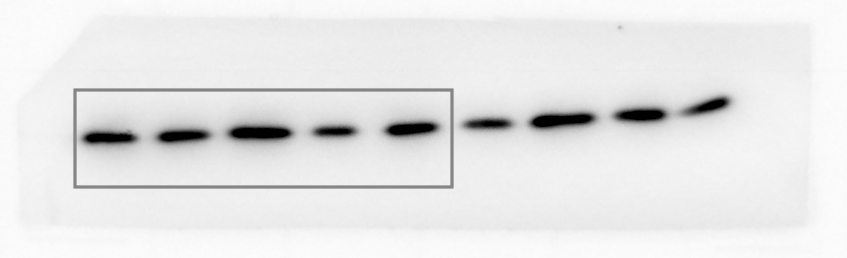

Supplement: Supplementary file 1 — Additional file 1. [file 12871_2022_1825_MOESM1_ESM.zip › Supplementary/MIF -original (mice).tif]

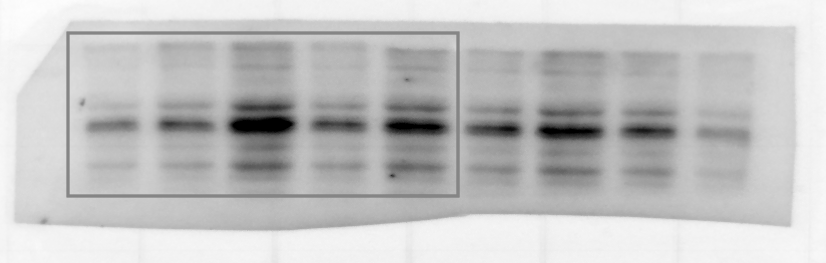

Supplement: Supplementary file 1 — Additional file 1. [file 12871_2022_1825_MOESM1_ESM.zip › Supplementary/p-AMPKa -original (mice).tif]

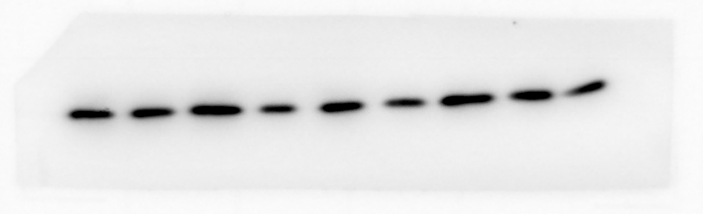
 **MIF**


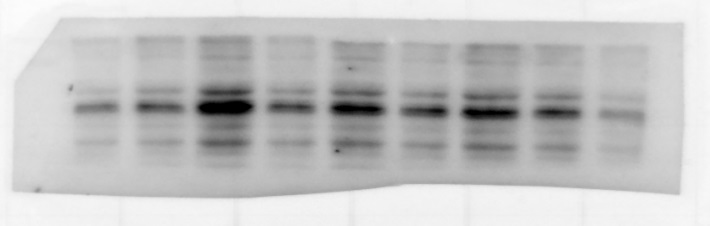
 **p-AMPKa**


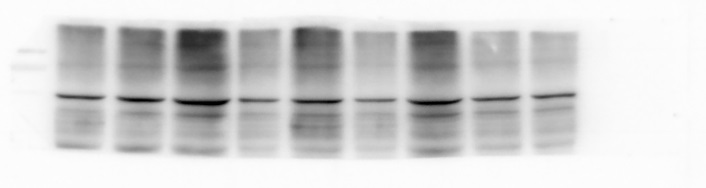
  **GLUT4**


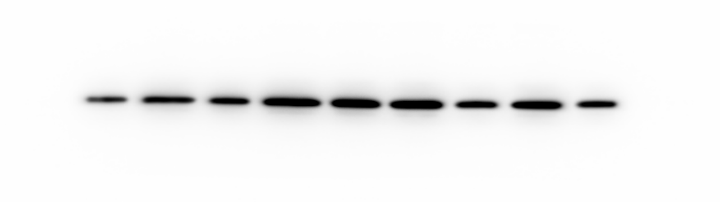
  **Bax**


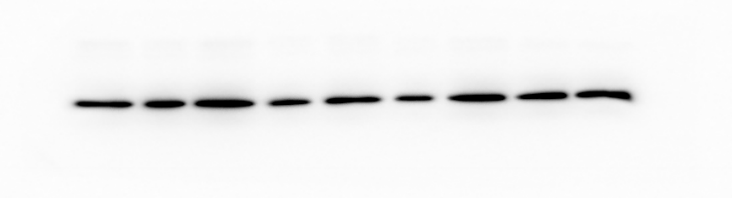
  **Bcl-2**


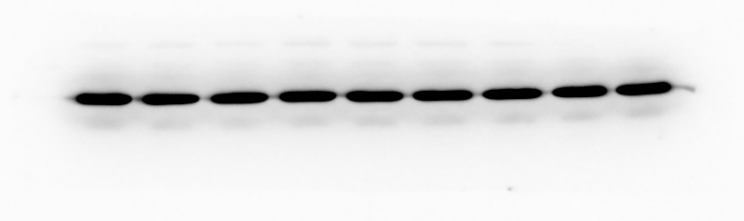
  **β-actin**

Supplementary figure 1. Western Blot full-length blots.

Supplement: Supplementary file 1 — Additional file 1. [file 12871_2022_1825_MOESM1_ESM.zip › Supplementary/Supplementary figure 1.docx]
